# LMBiS-Net: A lightweight bidirectional skip connection based multipath CNN for retinal blood vessel segmentation

**DOI:** 10.1038/s41598-024-63496-9

**Published:** 2024-07-02

**Authors:** Mufassir Matloob Abbasi, Shahzaib Iqbal, Khursheed Aurangzeb, Musaed Alhussein, Tariq M. Khan

**Affiliations:** 1 Department of Electrical Engineering, Abasyn University Islamabad Campus (AUIC), Islamabad, 44000 Pakistan; 2 Department of Computer Engineering, College of Computer and Information Sciences, King Saud University, Riyadh, P. O. Box 51178, 11543 Saudi Arabia; 3 School of Computer Science and Engineering, University of New South Wales, Sydney, NSW Australia

**Keywords:** Retina blood vessel segmentation, Bidirectional skip connections, Multipath connections, Macular degeneration, Retinal diseases

## Abstract

Blinding eye diseases are often related to changes in retinal structure, which can be detected by analysing retinal blood vessels in fundus images. However, existing techniques struggle to accurately segment these delicate vessels. Although deep learning has shown promise in medical image segmentation, its reliance on specific operations can limit its ability to capture crucial details such as the edges of the vessel. This paper introduces LMBiS-Net, a lightweight convolutional neural network designed for the segmentation of retinal vessels. LMBiS-Net achieves exceptional performance with a remarkably low number of learnable parameters (only 0.172 million). The network used multipath feature extraction blocks and incorporates bidirectional skip connections for the information flow between the encoder and decoder. In addition, we have optimised the efficiency of the model by carefully selecting the number of filters to avoid filter overlap. This optimisation significantly reduces training time and improves computational efficiency. To assess LMBiS-Net’s robustness and ability to generalise to unseen data, we conducted comprehensive evaluations on four publicly available datasets: DRIVE, STARE, CHASE_DB1, and HRF The proposed LMBiS-Net achieves significant performance metrics in various datasets. It obtains sensitivity values of 83.60%, 84.37%, 86.05%, and 83.48%, specificity values of 98.83%, 98.77%, 98.96%, and 98.77%, accuracy (acc) scores of 97.08%, 97.69%, 97.75%, and 96.90%, and AUC values of 98.80%, 98.82%, 98.71%, and 88.77% on the DRIVE, STARE, CHEASE_DB, and HRF datasets, respectively. In addition, it records F1 scores of 83.43%, 84.44%, 83.54%, and 78.73% on the same datasets. Our evaluations demonstrate that LMBiS-Net achieves high segmentation accuracy (acc) while exhibiting both robustness and generalisability across various retinal image datasets. This combination of qualities makes LMBiS-Net a promising tool for various clinical applications.

## Introduction

To diagnose various clinical conditions, it is vital to analyse the morphology and characteristics of retinal blood vessels, which are only observable by retinal imaging^1^. The study of retinal pathology provides valuable information on a range of ocular diseases^2,3^. The intricate structure of retinal blood vessels can be significantly altered by medical disorders such as hypertension and diabetic retinopathy, resulting in changes in the breadth and curvature of the vessels^4^. Due to the intricate relationship between retinal diseases and the ongoing changes in retinal vascular architecture, analysing the distribution of vessel density provides invaluable insights into various underlying medical conditions^5^. The development of the retinal vascular architecture and the distribution of vessel density is a multifaceted process shaped by genetic, environmental, and physiological elements. Angiogenesis, the formation of new blood vessels, initiates the establishment of a primary vascular plexus during retinal development^6^.

Consequently, early detection of associated disorders is highly dependent on segmentation of the retinal blood vessels by facilitating quantitative assessment of vascular alterations, helping in early diagnosis, risk assessment, and treatment supervision, ultimately enhancing patient outcomes. The complex and asymmetric architecture of the retinal blood vessels comes in a variety of forms, making the segmentation of these veins a challenging process^7^. There are two categories of current retinal vascular segmentation techniques: algorithmic segmentation and manual segmentation^8^. Due to the complexity of retinal vessels, manual segmentation requires ophthalmologists to manually design dynamic vessels. This method is time-consuming, expensive, and difficult. The level of experience of the physician has a significant impact on the precision of manual segmentation of the retinal blood vessels. However, because this method is subjective, various physicians will get different findings, which will impact the accuracy of the segmentation^9^. Consequently, early detection of associated disorders is highly dependent on segmentation of the retinal blood vessels.

To create an automated segmentation framework for retinal vessels, some limiting issues must be addressed. The extraction of feature representations depends critically on the quality of the fundus images^10,11^. Fundus images with low contrast have difficulties since it is more difficult to obtain structural details from the retina^12^. The complex and dynamic nature of retinal blood vessels makes it imperative to capture spatial information as efficiently as possible, especially when dealing with varying thicknesses of vessels^13^. Accurate extraction of slender vessels against intricate fundus image backgrounds requires the augmentation of high-level features. The quality of the segmented mask that is obtained is substantially affected by these key factors and elements. To establish a reliable and precise automated segmentation framework of retinal vessels, meticulous attention must be devoted to mitigating these constraining factors^14^.

In recent years, we have witnessed a rise in deep segmentation models inspired by the U-Net architecture^15^, aimed at improving segmentation precision and efficiency in medical image segmentation tasks. These models employ various optimisation schemes, such as attention mechanisms^16,17^, feature aggregation^18,19^, recurrent convolution^20^, hybrid networks^21^, etc., which results in promising performance.

Yan *et al*^22^ suggested a three-stage segmentation network that, in the first two stages, addresses the distinct segmentation issues for thick and thin vessels and, in the third stage, combines the characteristics of these stages. Yang *et al*^23^ introduced a hybrid fusion network to process thick and thin vessel information separately, with a subsequent fusion network to aggregate these two types of vessel information, achieving high recall on the DRIVE dataset. Wang *et al*^24^ presented a multidecoder structure to process different types of vascular information by classifying features as ‘Hard’ or ‘Easy’, and then processing them separately in subdecoders to adaptively handle different types of feature.

In another approach, a dual-path encoder^25^ was proposed, consisting of two paths that process spatial features and contextual features separately using different sizes of convolutional kernels to capture richer features. Staal *et al*^26^ introduced Bridge-Net, which utilized the U-Net architecture as the foundational network. It incorporated two patches at distinct scales to analyze vascular features, enabling larger-scale patches to supply additional contextual information to smaller-scale patches. DEF-Net^27^ used a dual encoder structure to enrich feature representation, capture detailed features and contextual features through CNN and RCNN structures, respectively, and facilitate feature fusion with multiscale fusion blocks.

Although these segmentation methods process thick and thin vessels individually based on their distinct characteristics, the multipath structure introduces an additional computational burden. Moreover, the features of the thin vessels are often lost in successive convolution and pooling operations, resulting in inefficient fusion of thick and thin vessel information and making it challenging to achieve the desired segmentation accuracy.

**Figure 1:**
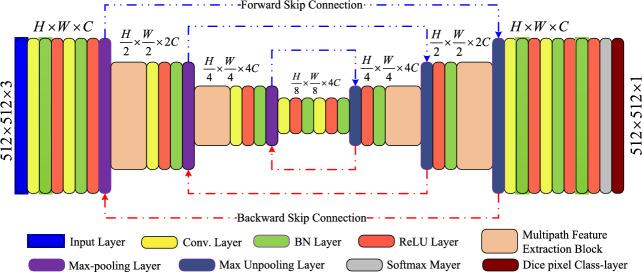
Architecture of the proposed LMBiS-Net. $H \times W \times C$ represents the height, width, and number of channels used at each convolution layer.

We present a novel lightweight convolutional neural network (CNN) with multipath bidirectional skip connections for the segmentation of vascular structures in retinal images, which aims to tackle the issues of the flow of gradients, aid in preserving fine-grained details, enable better object localisation, enhance robustness to data variations, accelerate convergence, and act as a form of regularisation to prevent overfitting. The proposed LMBiS-Net scheme, illustrated in Fig. 1, is based on a lightweight architecture. First, we present the feature extraction process using the multipath feature extraction block. Furthermore, the encoder-decoder design integrates robust feature fusion via bidirectional skip connections. Furthermore, to minimise the loss of spatial information, the encoder only uses two max-pooling layers. In our investigations, three different datasets created especially for the segmentation of retinal blood vessels were used. These tests proved that the suggested design works well. The following are the main benefits of LMBiS-Net:

1. We introduce LMBiS-Net, a lightweight convolutional neural network (CNN) specifically designed for retinal vessel segmentation. LMBiS-Net achieves exceptional performance with a remarkably low number of learnable parameters (only 0.172 M), making it highly efficient.
2. The design of the proposed LMBiS-Net incorporates bidirectional skip connections for better gradient flow, feature reuse, and efficient information flow between the encoder and decoder.
3. To further enhance computational efficiency, we employed a filter selection strategy that minimizes filter overlap. This optimization significantly reduces training time while maintaining accuracy.
4. To thoroughly assess LMBiS-Net’s robustness and generalizability, we conducted comprehensive evaluations across diverse retinal image datasets.

The manuscript is organised into six sections. Section "Introduction" explains the issue of retinal blood vessel segmentation and justifies a novel solution. Section "Literature review" explains the relevant research in the area of retinal blood vessel segmentation, emphasising the shortcomings of current techniques and the demand for a new strategy. In Section "Proposed method", a detailed explanation of the proposed approach is provided, outlining all its essential elements and workings, including the encoder-decoder network architecture. Section "Experimental setup" presents the experimental setup used for the experimentation. Section "Results and discussions" gives a detailed analysis of the performance assessment carried out on three benchmark datasets that are available to the public, together with a computational complexity analysis of the results. Finally, Section "Conclusions" summarises the main findings of the suggested approach and its prospects for advancement in the field of retinal blood vessel segmentation in conclusion.

## Literature review

### Retinal blood vessels segmentation networks

Retinal blood vessel segmentation has been addressed through various methodologies in the existing literature. In some studies, ensemble decision tree classifiers^28^ were utilised for image analysis, while others used wavelet-based frameworks^10,29^ to approximate the diameter of the retinal vessel in fundus images. A deep learning model named Sine-Net^30^ was presented for the segmentation of retinal vessels. FCNs have emerged as a common choice for semantic segmentation in this context, with the M3FCN, a variant that incorporates a multiscale input block, as described in^31^. A comprehensive comparative examination of the models of segmentation of DL/ML-based retinal vessels is detailed in^32^. Chen *et al*. extended this concept in^33^ to introduce Deeplab V3+, an enriched version that encompasses both encoder and decoder components, facilitating the generation of segmented masks. In a different vein, ResDo-UNet, as presented in^34^, enhances the U-Net model by incorporating a residual network as the backbone. This modification enhances context capture for retinal vessel segmentation. Similarly, the work of^35^ uses an image quality enhancement strategy in conjunction with a CNN-based method to segment retinal vessels in fundus images, improving the accuracy of the segmented masks that are produced. Moreover, the research conducted in^36^ introduces a GAN-based model. In order to create segmented masks and carry out binary classification, this model uses a dual architecture made up of a generator and discriminator. These many approaches are representative of the ongoing investigation and development of image segmentation methods, with special attention to the segmentation of the retinal arteries. These developments contribute significantly to increasing the efficacy of medical analyses and diagnoses.

**Figure 2:**
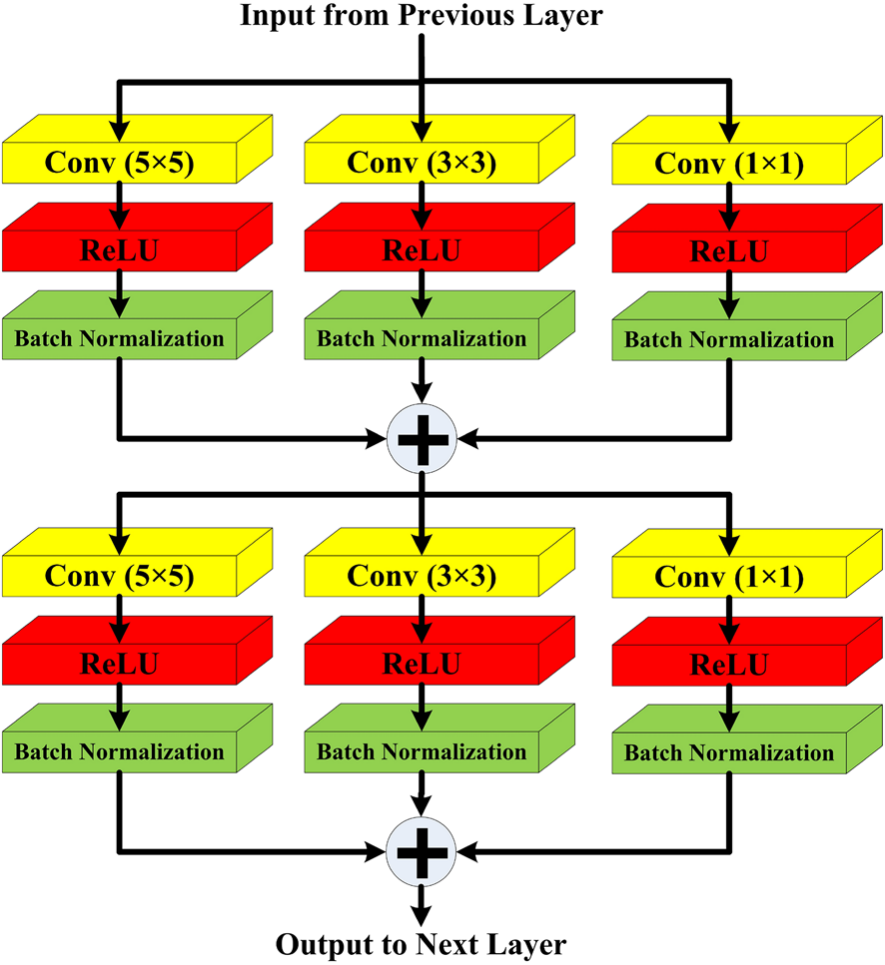
Schematic of the proposed multipath block used for the feature information extraction. The proposed block uses consecutive multipath blocks for feature extraction.

### Lightweight segmentation networks

The lightweight design of semantic segmentation networks has increasingly become a hot topic in image segmentation challenges, attracting the attention of many experts in recent years. A lightweight model based on a double branch structure called BiSeNet^37^ was developed. It uses several paths to extract spatial and semantic information. In addition, other models use common components to save computing time. DMFNet^38^ separates channels into numerous groups and employs weighted three-dimensional extended convolution to minimise parameters and increase the inference efficiency of the model. Deep separable convolution was used by Xception^39^ and MobileNets^40,41^ to effectively enhance inference speed. The Inception lightweight backbone and the dense module are combined in the Dense-Inception U-Net^42^ to extract high-level semantic data with a lightweight encoder. Compared to more complex models, ShuffleNets^43^ uses group convolution and channel shuffling, significantly reducing computational cost. However, lightweight segmentation networks are less effective for somewhat complicated medical images than they are for natural images^44,45^. Some researchers have also created lightweight segmentation networks for medical images. However, few medical image segmentation networks can achieve both low complexity and high inference speed while ensuring performance. U-Net++^46^ uses minimal parameters to obtain high segmentation performance while ignoring the model’s inference time. Using depth- and point-wise convolution, a lightweight V-Net^47^ ensures segmentation accuracy and uses fewer parameters, but it does not speed up the model’s inference process. PyConvU-Net^48^ improves segmentation accuracy and uses fewer parameters by swapping out all of the U-traditional Net convolution layers for pyramidal convolution. However, PyConvU- Net’s^48^ inference speed is slow.

## Proposed method

### Network structure

The proposed LMBiS-Net consists of bottleneck layers, a multipath block, three encoder blocks, and three decoder blocks arranged in a systematic manner as shown in Fig. 1. The following are the specifics of each component: ReLU activation functions and convolution layers are utilised to extract features from the input. In order to improve training stability and speed up convergence during optimisation, a batch normalisation layer is used. After batch normalisation, max pooling is used to reduce the spatial dimensions of the feature maps while retaining significant feature information. The multipath feature extraction block incorporates convolution blocks $1\times 1$, $3\times 3$, and $5\times 5$, each followed by ReLU activation and batch normalisation. The outcome of this encoding step is manifested as a feature map, which is generated by aggregating the outputs of these individual blocks. Capturing computational efficiency during model training is an essential role of the bottleneck layer. Usually, it serves as a link between the parts that encode and decode data. Transpose convolution layers are utilised for up-sampling at the decoder step, which effectively increases the spatial dimensions of the feature maps. The opposite of ordinary convolution is called transpose convolution. A softmax layer is used for scale invariance and normalisation in the last decoder step. It generates the output of the model in a probabilistic format. By employing dice-based pixel categorization, the overlap between the actual image and the anticipated image is calculated. It is often used in segmentation tasks to assess the degree of similarity between predicted and actual segmentations. In neural networks, skip connections are a potent architectural feature that facilitates deep model training. By integrating bidirectional skip connections across its encoding and decoding layers, the proposed LMBiS-Net improves gradient flow, feature reuse, enhanced localisation, robustness to input variations, and faster convergence. Furthermore, reverse skip connections are used to remap the decoded features to the encoder.

### Multipath feature extraction block

A multipath feature extraction block is introduced in the proposed LMBiS-Net to process information through multiple parallel pathways. The key advantage of a multipath feature extraction block is its ability to introduce feature diversity. Within the block, every pathway has the ability to focus on extracting unique characteristics from the incoming data. Because of this diversity, the network is able to learn various patterns or details, leading to a more thorough and diversified representation of the input. By processing the input data through multiple paths with varying levels of complexity, a multipath feature extraction block helps to build a hierarchical representation. This is important for vascular segmentation tasks, as it implies that the network can capture both low-level characteristics (such as edges and textures) and high-level features (such as complex objects or structures). As a kind of regularisation, multipath feature extraction blocks promote variation among paths. By lowering the possibility that the whole network will memorise the training set, this variety aids in the prevention of overfitting. It promotes robustness and generalisation. The proposed multipath feature extraction block is shown in (Fig. 2) and performs the following operations: The convolutions $1\times 1$, $3\times 3$, and $5\times 5$ are applied to the input data. The ReLU activation function is applied to introduce non-linearity. Batch normalisation is performed to stabilise and accelerate training. The output of batch normalisation in all convolution blocks is aggregated, resulting in the generation of intermediate outputs. The output of one step becomes the input of the next, and this process is repeated in successive phases. The bottleneck layer receives the final output and is responsible for dimensionality reduction, improving parameter efficiency, facilitating increased depth, and improving memory efficiency. The input $I_{in}$ is passed through a convolutional block of three different scales, s, and the intermediate output $S_{1}$ is obtained using equation 1.

1 $$\begin{aligned} S_{1}=\sum _{k=1}^{3}\beta \left( \text {ReLU} \left( f^{n\times n} \left( I_{in} \right) \right) \right) \end{aligned}$$

where $\beta$ is the batch normalisation operation, $\text {ReLU}$ is the activation function, $f^{n\times n}$ is the kernel size convolution operation $n\times n$, and $n=2k-1$.

2 $$\begin{aligned} I_{out}=\sum _{k=1}^{3}\beta \left( \text {ReLU} \left( f^{n\times n} \left( S_{1} \right) \right) \right) \end{aligned}$$

The output $I_{out}$ of the multipath feature extraction block is calculated using equation 2.

**Table 1:** Description of the datasets used for experimentation and evaluation of the proposed LMBiS-Net.

Dataset	Number of Images			Train/Test	Augmented	Image	Resized	FOV	Image
	Training	Testing	Total	Split	Images	Resolution	Resolution		Format
DRIVE	20	20	40	N. A	760	$584 \times 565$	$512\times 512$	$45^{o}$	.tif
STARE	20	–	20	leave-one-out	608	$605 \times 700$	$512\times 512$	$35^{o}$	.ppm
CHASE	28	–	28	leave-one-out	760	$990 \times 960$	$512\times 512$	$30^{o}$	.jpg
HRF	36	9	45	N. A	855	$3504 \times 2336$	$512\times 512$	$60^{o}$	.jpg

**Table 2:** Ablation study performed on DRIVE dataset. $\uparrow$ shows that the higher values are better whereas $\downarrow$ shows that the lower values are better.

Method	Param (M) $\downarrow$	$\frac{T_{training}}{epoch} in (min)$ $\downarrow$	Performance Measures (%)					
			$S_{e} \uparrow$	$S_{p} \uparrow$	$A_{cc} \uparrow$	$F_{1}-Score \uparrow$	$AUC \uparrow$	$J_{index} \uparrow$
Base Line (BL)	13.00	6.46	80.54	98.06	95.33	79.53	97.12	63.94
Lightweight Base Line(LBL)	0.095	3.20	78.10	98.18	96.42	79.21	88.14	62.44
LBL + Multipath feature extraction block (MFEB)	0.150	1.98	80.35	98.28	96.70	81.00	89.31	64.35
LBL+ MFEB + Bidirectional skip connections	0.172	2.06	83.60	98.32	96.83	81.76	89.81	65.33

**Table 3:** Performance Evaluation of the proposed LMBiS-Net on different annotations marked by different experts.

Dataset	Annotations	Performance measure (%)				
		$S_{e} \uparrow$	$S_{p} \uparrow$	$A_{cc} \uparrow$	$F_{1}-Score \uparrow$	$AUC \uparrow$
DRIVE^49^	1st Manual	83.60	98.83	97.08	83.43	98.80
	2nd Manual	88.82	97.88	98.29	88.65	99.00
	**Average**	**86.21**	**98.36**	**97.69**	**86.04**	**98.90**
CHASE_DB1^50^	1st Observer	86.05	98.96	97.75	83.54	98.71
	2nd Observer	93.27	98.81	98.96	90.76	98.91
	**Average**	**89.66**	**98.89**	**98.36**	**87.15**	**98.81**
STARE^51^	Dr. Adam Hoover	84.37	98.77	97.69	84.44	98.82
	Dr. Valentina Kouznetsova	91.59	98.62	98.90	91.66	99.02
	**Average**	**87.98**	**98.70**	**98.30**	**88.05**	**98.92**

**Figure 3:**
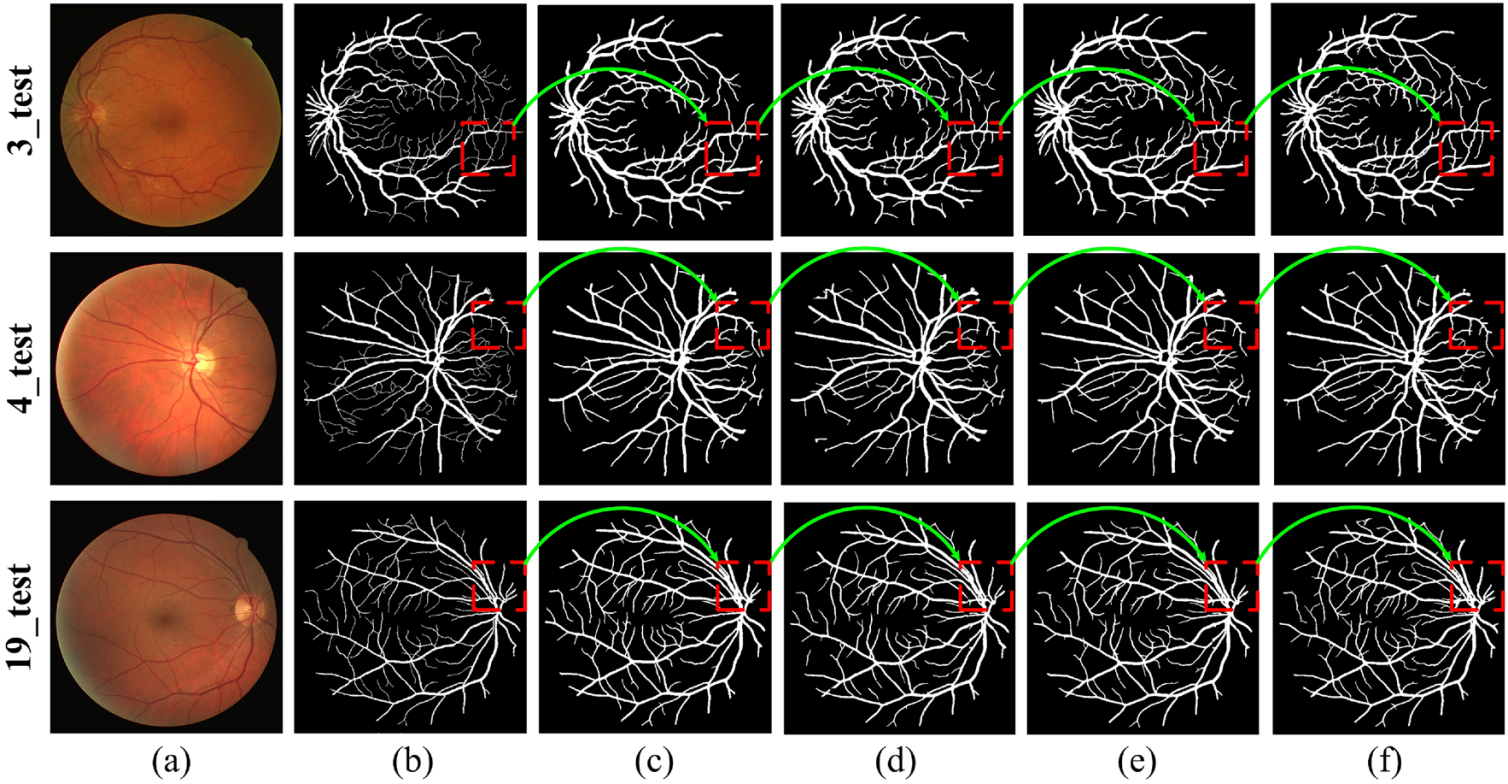
Small Vessel detection with different paths of the multipath feature extraction block: (**a**) Input image, (**b**) Corresponding ground truth, (**c**) Results of lightweight baseline with $5 \times 5$ path, (**d**) Results of lightweight baseline with $3 \times 3$ path, (**e**) Results of lightweight baseline with $3 \times 3$ and $5 \times 5$ paths, and (**f**) Results of lightweight baseline with $5 \times 5$, $3 \times 3$, and $1 \times 1$ paths.

**Table 4:** This Table compared LMBiS-Net to numerous other approaches using the DRIVE^49^ dataset. The optimal outcomes are highlighted in bold. The performance of alternative approaches is derived from the referenced academic works. Unreported findings are denoted by a hyphen. $\uparrow$ shows that the higher values are better.

Method	Performance measures in (%)				
	$S_{e} \uparrow$	$S_{p} \uparrow$	$A_{cc} \uparrow$	$AUC \uparrow$	$F_{1} \uparrow$
Att UNet^52^	79.46	97.89	95.64	97.99	82.32
BCDU-Net^53^	79.84	98.03	95.75	98.11	82.49
Bio-Net^54^	82.20	98.04	96.09	82.06	98.26
CTF-Net^55^	78.49	98.13	95.67	97.88	82.41
CSU-Net^25^	80.71	97.82	95.65	98.01	82.51
OCE-Net^56^	80.18	98.26	95.81	98.21	83.02
G-Net Light^57^	81.92	98.29	96.86	-	82.02
LDMRes-Net^58^	83.58	98.32	97.02	98.51	83.09
**Proposed LMBiS-Net**	**83.60**	**98.83**	**97.08**	**98.80**	**83.43**

## Experimental setup

### Datasets description

Three retinal fundus image datasets that are available to the public were utilised to assess and contrast the proposed model with competing models. DRIVE, STARE, CHASE_DB1, and HRF. (Table  1) provides an overview of these freely accessible datasets, which are used in the study for comparisons and experiments.

**Figure 4:**
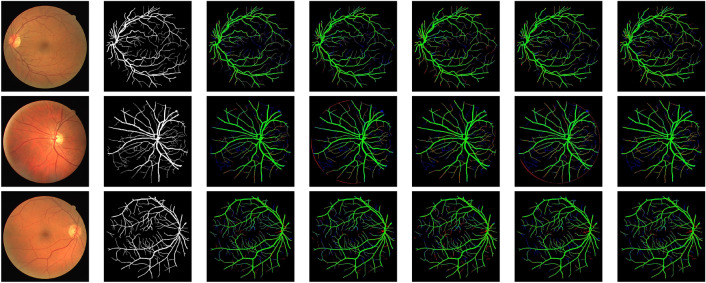
LMBiS-Net segmentation results and comparison techniques applied to exemplar test images from the DRIVE dataset. From left to right: test images, ground truths, and segmentation results for BCDU-Net, MultiResNet, SegNet, Unet++, and our proposed LMBiS-Net, respectively. False negative pixels are shown in blue, false positives in red, and true positives in green.

### Evaluation criteria

The segmentation results were compared with the associated ground-truth photos to determine the accuracy of the segmentation findings. Every pixel in the final image was labelled as a properly segmented foreground pixel ($T_{P}$) or a background pixel ($T_{N}$) or as a pixel that was improperly segmented foreground ($F_{P}$) or background ($F_{N}$).

Sensitivity (Se) measures the proportion of correctly identified positives ($T_{P}$) among all actual positives ($T_{P}$ + $F_{N}$). In simpler terms, it reflects the method’s ability to correctly detect all relevant objects (e.g., blood vessels) in the image. It can be expressed as:

3 $$\begin{aligned} S_{e}=\frac{T_{P}}{T_{P}+F_{N}} \end{aligned}$$

Complementary to Sensitivity (Se), Specificity (Sp) assesses a method’s ability to correctly identify negative samples (e.g., background pixels) in an image. It calculates the proportion of true negatives ($T_{N}$) among all actual negatives ($T_{N}$ + $F_{P}$). A higher Sp value indicates better performance in correctly classifying true background pixels and avoiding false positives. Mathematically, it can be represented as:

4 $$\begin{aligned} S_{p}=\frac{T_{N}}{T_{N}+F_{P}} \end{aligned}$$

Accuracy (Acc) measures the overall effectiveness of a segmentation method by considering the proportion of correctly classified pixels. It takes into account both true positives ($T_{P}$ + $T_{N}$) to all pixels in the image. It reflects the overall effectiveness of the method. The formula for accuracy can be expressed as follows:

5 $$\begin{aligned} A_{cc}=\frac{T_{P}+T_{N}}{T_{P}+T_{N}+F_{P}+F_{N}} \end{aligned}$$

The $F_{1}$-score, also known as the Dice similarity coefficient (DSC), is a popular metric used to evaluate the performance of a classification model. It takes into account both precision and recall, aiming to strike a balance between them, and can be expressed as:

6 $$\begin{aligned} F_{1}-Score =\frac{2 \times T_{P}}{(2\times T_{P})+F_{P}+F_{N}} \end{aligned}$$

The area under the curve (AUC) is a metric used in receiver operating characteristic (ROC) analysis to assess a model’s ability to distinguish between positive and negative classes. The ROC curve itself plots the true positive rate (TPR) against the false positive rate (FPR) at various classification thresholds. Calculating the AUC involves integrating the curve formed by the product of (1 + TPR) and (1 - FPR) over different thresholds. This value summarises the model’s overall performance in discriminating between positive and negative samples.

7 $$\begin{aligned} AUC=1-\frac{1}{2}\left( \frac{F_{P}}{F_{P}+T_{N}}+\frac{F_{N}}{F_{N}+T_{P}}\right) \end{aligned}$$

**Table 5:** This Table compared LMBiS-Net to numerous other approaches using STARE^51^ dataset. The optimal outcomes are highlighted in bold. The performance of alternative approaches is derived from the referenced academic works. Unreported findings are denoted by a hyphen. $\uparrow$ shows that the higher values are better.

Method	Performance measures in (%)				
	$S_{e} \uparrow$	$S_{p} \uparrow$	$A_{cc} \uparrow$	$AUC \uparrow$	$F_{1} \uparrow$
Att UNet^52^	77.09	98.48	96.33	97.00	-
BCDU-Net^53^	78.92	98.16	96.34	98.43	82.30
CC-Net^59^	80.67	98.16	96.32	98.33	81.36
OCE-Net^56^	80.12	98.65	96.72	98.76	83.41
Wave-Net^60^	79.02	98.36	96.41	-	81.40
G-Net Light^57^	81.70	98.53	97.30	-	81.78
LDMRes-Net^58^	84.07	98.75	97.64	98.72	84.24
**Proposed LMBiS-Net**	**84.37**	**98.77**	**97.69**	**98.82**	**84.44**

**Figure 5:**
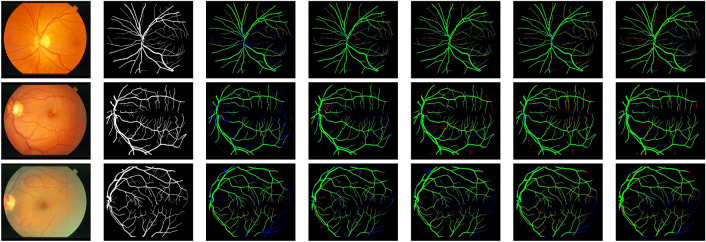
LMBiS-Net segmentation results and comparison techniques applied to exemplar test images from the STARE dataset. From left to right: test images, ground truths, and segmentation results for BCDU-Net, MultiResNet, SegNet, Unet++, and our proposed LMBiS-Net, respectively. False negative pixels are shown in blue, false positives in red, and true positives in green.

### Implementation and training

The proposed LMBiS-Net’s training specifics are outlined in this part using three benchmark datasets (DRIVE, STARE, and CHASE_DB). All images are standardized to a resolution of $512\times 512$ pixels to ensure consistency in size. After resizing, the images go through improvement processes, such as contrast modifications (with factors of [$\times 0.9, \times 1.1$]) and rotation at $10^{o}$. The size of the dataset increases ($38\times$) of the actual size of the datasets after the augmentations. Dice loss is used to train segmentation models. With an initial learning rate of 0.001 and a ceiling of 50 iterations, the Adam optimizer is employed. If there has been no improvement in performance on the validation set after seven epochs, half of the learning rate is removed. The early pausing strategy is employed to avoid overfitting. Using TensorFlow as the back-end, Keras implements the LMBiS-Net, which is trained on an NVIDIA K80 GPU.

### Ablation study on DRIVE dataset

The proposed LMBiS-Net is subjected to an ablation investigation using the DRIVE^49^ dataset. Experiments are conducted in a progressive manner. We started with implementing a single path encoder-decoder Unet^61^ network. Next, we reduced the number of parameters to produce a lightweight version of Unet by reducing the number of filters at each convolution layer and the encoder depth by minimising the pooling operations. Performance initially suffers when this value is decreased; however, this is later reduced and even improved by adding a multipath feature extraction block (MFEB). Subsequently, in the reconstruction stage of the suggested LMBiS-Net, bidirectional skip connections are used between the encoder and the decoder to enhance the information flow and refine the derived feature. Table 2 demonstrates the quantitative findings of the ablation study conducted, and it is clear that despite having relatively few learnable parameters, the performance is greatly enhanced. An ablation on the multipath feature extraction block is also carried out. Figure 3 presents the visual improvements made by each path and it is evident from the highlighted regions of Figure 3 that the multipath feature extraction block accurately detects the small vessels.

## Results and discussions

In this section, we have evaluated the performance of the proposed LMBiS-Net. To confirm the effects of our suggested improvements and evaluate the efficacy of each network block, we will first perform ablation experiments. Second, we have evaluated the performance of the proposed LMBiS-Net on different expert-marked annotations for the DRIVE, STARE, and CHASE_DB1 datasets. Details of the evaluations are given in (Table 3). It is important to mention that all comparisons with other state-of-the-art methods are made on the annotations provided by the $1^{st}$ observer for the DRIVE^49^ and CHASE_DB1^50^ dataset, whereas the annotations provided by Dr. Adam Hoover were used for the STARE^51^ dataset. Thirdly, we have contrasted our network’s segmentation outcomes using a number of cutting-edge methods. We havealso show visuvisualisationults to show how well our method can extract relevant patterns and features from the input data. Lastly, we have presented a computational complexity study of the suggested LMBiS-Net.

**Table 6:** This Table compared LMBiS-Net to numerous other approaches using the CHASE_DB^50^ dataset. The optimal outcomes are highlighted in bold. The performance of alternative approaches is derived from the referenced academic works. Unreported findings are denoted by a hyphen. $\uparrow$ shows that the higher values are better.

Method	Performance measures in (%)				
	$S_{e} \uparrow$	$S_{p} \uparrow$	$A_{cc} \uparrow$	$AUC \uparrow$	$F_{1} \uparrow$
Att UNet^52^	80.10	98.04	96.42	98.40	80.12
BCDU-Net^53^	77.35	98.01	96.18	98.39	79.32
OCE-Net^56^	81.38	98.24	96.78	98.72	81.96
G-Net Light^57^	82.10	98.38	97.26	-	80.48
LDMRes-Net^58^	85.95	98.88	97.55	98.61	81.94
**Proposed LMBiS-Net**	**86.05**	**98.96**	**97.75**	**98.71**	**83.54**

### LMBiS-net performance evaluation and comparison with state-of-the-art methods

We present a comparative analysis of the proposed LMBiS-Net, evaluating its segmentation efficacy in several datasets of retinal vascular segmentation.

This study employs four well-established datasets for segmentation of the retinal vessels: DRIVE^49^, STARE^51^, CHASE_DB1^50^, and HRF^62^. These datasets provide diverse images to evaluate the robustness and generalisability of LMBiS-Net. For a comprehensive performance assessment, we compare LMBiS-Net to established deep learning architectures: U-Net^63^ and SegNet^64^. This comparison with widely used benchmarks in the image segmentation community allows us to thoroughly contextualize LMBiS-Net’s performance advantages.

The quantitative assessment of LMBiS-Net is shown with numerous different approaches in Tables 5, 6, and 4. According to the tables, LMBiS-Net performs better than all other methods in terms of *F*1 score, area under the curve (AUC), sensitivity (Se), specificity (Sp) and accuracy (acc). Within the DRIVE dataset^49^, LMBiS-Net attained a sensitivity (Se) of 83.60%, specificity (Sp) of 98.83%, precision of 97.08%, area under the curve (AUC) of 98.80%, and *F*1 score of 83.4%, respectively. Similarly, LMBiS-Net achieved values of 84.37%, 98.77%, 97.69%, 98.82%, and 84.44% for sensitivity (Se), specificity (Sp), precision, AUC, and *F*1 score on the STARE dataset^51^, respectively. LMBiS-Net obtained scores of 86.05%, 98.96%, 97.75%, 98.71%, and 82.04% for sensitivity (Se), specificity (Sp), precision, AUC, and *F*1 score on the CHASE_DB1 dataset, respectively. An analysis of LMBiS-Net and other current approaches on the HRF dataset is presented in Table 7^62^. By attaining the best accuracy (acc), the area under the curve (AUC), and the dice scores of 78.73%, 88.77%, and 96.90%, respectively, LMBiS-Net surpasses all other approaches. These findings show that LMBiS-Net is superior to other techniques in precisely classifying retinal vessels in a variety of datasets and suggest its potential for reliable performance in real-world applications.

**Figure 6:**
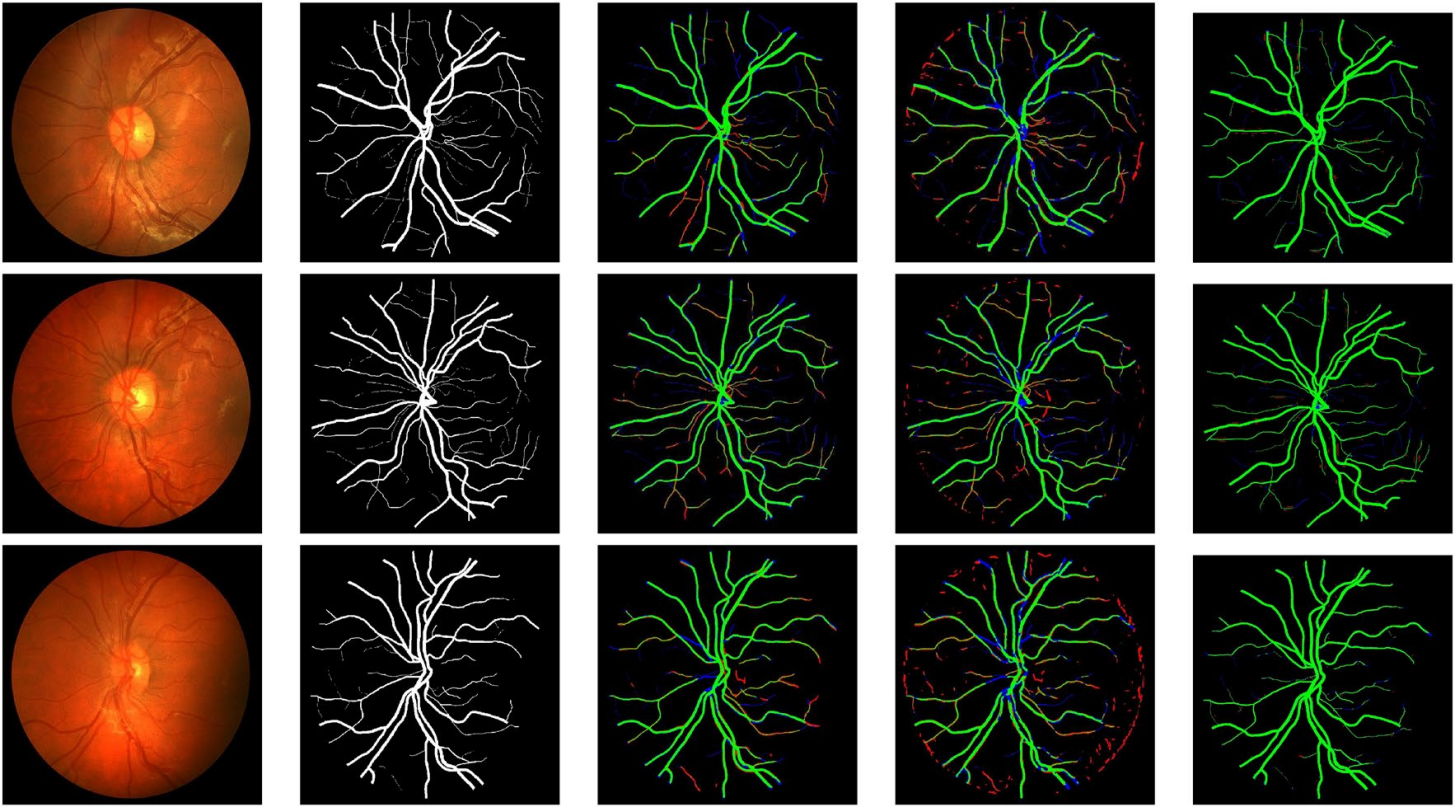
LMBiS-Net segmentation results and comparison techniques applied to exemplar test images from the CHASE_DB dataset. From left to right: test images, ground-truths, and segmentation results for BCDU-Net, MultiResNet, SegNet, Unet++, and our proposed LMBiS-Net, respectively. False negative pixels are displayed in blue, false positives in red and true positives in green.

We present a qualitative comparison of LMBiS-Net’s output with other techniques on the DRIVE, STARE, and CHASE datasets. This analysis complements our quantitative findings. As shown in Fig. 4, LMBiS-Net outperforms other approaches in reducing false positives in tiny vessels. For instance, U-Net variations produce more false positives since they aren’t good at accurately delineating vessel boundaries. Similarly, other systems such as SegNet and BCDU-Net have issues with false positive rates or poor accuracy when it comes to gathering vessel details. In contrast, LMBiS-Net achieves better segmentation by collecting important vessel data with few false positives. As seen in Fig. 5, alternative approaches often result in a higher number of false positives, especially in the vicinity of tiny arteries, optic nerves, and intricate retinal structures. The complexity of the dataset is probably to blame for this. With a low false positive rate and improved resistance to these artefacts, LMBiS-Net is able to preserve intricate vessel details. The results indicate that LMBiS-Net can efficiently handle tough conditions, which highlights its promise in this field. When the CHASE_DB1 dataset is processed using LMBiS-Net, comparable patterns are noted (Fig. 6). The approach consistently achieves precise vessel segmentation in the face of obstacles such as fluctuations in image quality and abnormalities in the vessels. No matter how low the contrast or how many overlapping vessel patterns there are, LMBiS-Net is able to successfully minimise false positives while maintaining actual vessel structures. These findings provide more evidence of the effectiveness and resilience of the method in a variety of diverse datasets and image conditions.

**Table 7:** Comparative analysis of LMBiS-Net’s performance against recent methodologies on the HRF dataset^62^. The optimal outcomes are highlighted in bold. The performance of alternative approaches is derived from the referenced academic works. Unreported findings are denoted by a hyphen. $\uparrow$ shows that the higher values are better.

Model	Performance measures (%)				
	$S_{e} \uparrow$	$S_{p} \uparrow$	$A_{cc} \uparrow$	$AUC \uparrow$	$F_{1} \uparrow$
U-Net^61^	–	–	95.87	83.05	72.39
SA-U-Net^65^	–	–	95.64	82.70	71.18
CogSeg^66^	–	–	96.22	84.31	74.75
SuperVessel^67^	–	–	96.54	85.06	76.74
Bio-Net^54^	82.09	98.34	96.79	85.60	76.26
**Proposed LMBF-Net**	**83.48**	**98.77**	**96.90**	**88.77**	**78.73**

**Table 8:** Cross validation of the proposed LMBiS-Net across the DRIVE, STARE, and CHASE_DB datasets. $\uparrow$ shows that the higher values are better.

Training dataset	Testing dataset	Method	Performance measures in (%)				
			$S_{e} \uparrow$	$S_{p} \uparrow$	$A_{cc} \uparrow$	$AUC \uparrow$	$F_{1} \uparrow$
DRIVE	CHASE_DB	BCDU-Net^53^	72.17	98.20	94.86	93.27	73.67
		G-Net Light^57^	72.92	98.15	95.02	97.09	73.13
		Att UNet^52^	75.36	98.62	94.65	97.32	75.22
		**LMBiS-Net**	**79.02**	**98.70**	**94.95**	**97.46**	**78.56**
	**STARE**	BCDU-Net^53^	74.99	97.98	95.63	96.21	75.06
		G-Net Light^57^	77.22	97.15	95.48	94.86	77.98
		Att UNet^52^	75.06	98.28	95.53	96.23	76.89
		**LMBiS-Net**	**79.56**	**98.31**	**95.89**	**97.06**	**80.22**
STARE	DRIVE	BCDU-Net^53^	75.34	97.23	95.07	93.27	76.87
		G-Net Light^57^	76.77	97.15	95.21	95.32	77.02
		Att UNet^52^	75.36	98.03	95.68	96.07	76.23
		**LMBiS-Net**	**77.38**	**98.70**	**95.79**	**97.46**	**78.01**
	CHASE_DB	BCDU-Net^53^	77.29	97.65	94.88	95.09	78.25
		G-Net Light^57^	76.22	96.99	94.76	95.24	77.04
		Att UNet^52^	77.01	97.29	94.03	96.11	77.54
		**LMBiS-Net**	**78.99**	**98.02**	**95.66**	**96.92**	**78.52**
CHASE_DB	DRIVE	BCDU-Net^53^	72.17	98.20	94.86	93.27	73.32
		G-Net Light^57^	72.92	98.15	95.02	97.09	74.59
		Att UNet^52^	75.36	98.62	94.65	97.32	75.99
		**LMBiS-Net**	**78.82**	**98.44**	**95.55**	**97.34**	**79.07**
	STARE	BCDU-Net^53^	74.99	97.98	95.63	96.21	75.01
		G-Net Light^57^	71.88	98.16	95.48	94.86	72.03
		Att UNet^52^	75.06	98.28	95.53	96.23	76.11
		**LMBiS-Net**	**76.19**	**98.45**	**95.71**	**96.82**	**76.22**

We conducted cross-validation studies on the DRIVE, STARE, and CHASE_DB datasets to evaluate the generalisability of our proposed LMBiS-Net and compared the findings with other cutting-edge techniques. Performance metrics, including sensitivity ($S_{e}$), specificity (*S* *p*), area under the curve (*AUC*), $F_{1}$, and accuracy ($A_{cc}$), were evaluated. As depicted in Table 8, when compared to alternative approaches, the suggested LMBiS-Net performs the best on all performance metrics. These results show that the suggested LMBiS-Net, which has very few learnable parameters, has greater generalisability.

In summary, our comprehensive analysis demonstrates that LMBiS-Net surpasses other methods in both accuracy (acc) and robustness. Quantitative results consistently highlight the strong performance of LMBiS-Net, while visual comparisons emphasise its ability to accurately capture retinal vascular architecture with minimal false positives. These findings provide substantial evidence of the efficacy of our proposed approach for vascular segmentation tasks in the retinal images and its potential to aid in the detection and tracking of retinal disorders.

### Computational complexity analysis of the LMBiS-net

Table 9 provides a detailed examination of the parameters and size of the model. One of the most coveted goals in the field of deep learning-based medical image analysis is to achieve good performance with fewer parameters. This is good since it consumes fewer computer resources and produces shorter training and inference periods. In particular, the model with the lowest parameter count is highlighted in bold in Table 9. The analysis demonstrates that LMBiS-Net outperforms other models while using a minimal number of parameters (only 0.172 M). This demonstrates its usefulness and efficiency when used in the segmentation of retinal vessels. This finding has great potential to improve automated diagnostics of retinal diseases and raise the bar for medical treatment. LMBiS-Net may make it possible for medical professionals to analyse retinal images more quickly and accurately, which would help to identify and treat early retinal problems. The effectiveness of the model in achieving excellent segmentation results with a reduced parameter count opens up new avenues for enhancing medical image analysis and patient care in the field of ophthalmology.

**Table 9:** Computational requirements comparisons of the LMBiS-Net with state-of-the-art methods. $\downarrow$ shows that the lower values are better.

Method	Param (M) $\downarrow$	Size (MB) $\downarrow$	$\frac{T_{training}}{epoch} in (min)$ $\downarrow$	$\frac{T_{inference}}{image} in (msec)$ $\downarrow$
MobileNet-V3-small^68^	2.50	11	5	30.32
MultiRes UNet^69^	7.20	43.5	8	55.60
VessNet^70^	9.30	36.6	8.2	60.34
M2U-Net^71^	0.55	2.20	3.3	20.25
G-Net Light^57^	0.39	1.52	3.01	17.86
**Proposed LMBiS-Net**	**0.172**	**0.56**	**2.06**	**16.32**

## Conclusions

With just 0.172 million learnable parameters, the lightweight multipath bidirectional skip connection-based network is the retinal image segmentation model that we have suggested in this study. The multipath feature extraction block included in the proposed LMBiS-Net greatly increases the computational efficiency of the network. In order to prevent feature overlap, the suggested network employs the ideal number of filters. This significantly reduces training time. The proposed network includes bidirectional skip links to adjust the encoder properties and enhance the information flow between the encoder and the decoder. The durability and adaptability of the proposed network are confirmed by the experimental results of the LMBiS-Net on many retinal image datasets of retinal blood vessels. LMBiS-Net outperforms all other methods for segmentation of the retinal blood vessels using a small number of learnable parameters, which is beneficial for the early detection and management of retinal diseases. Although the proposed LMBiS-Net has improved performance compared to other state-of-the-art methods, the performance is compromised on the small vessel segmentation on high-resolution images due to resizing effects. In the future, the issue of small vessels can be addressed by emphasizing the particular region through attention mechanisms.

## Data Availability

The datasets used during the current research work are available in the [DRIVE; CHASE_DB1; STARE] repositories, [https://www.kaggle.com/datasets/andrewmvd/drive-digital-retinal-images-for-vessel-extraction; https://www.kaggle.com/datasets/rashasarhanalharthi/chase-db1;https://www.kaggle.com/datasets/vidheeshnacode/stare-dataset/code].
